# Urinary pH is an independent predictor of upper tract recurrence in non-muscle-invasive bladder cancer patients with a smoking history

**DOI:** 10.1038/s41598-021-00184-y

**Published:** 2021-10-19

**Authors:** Hiroki Ide, Eiji Kikuchi, Koichiro Ogihara, Naoya Niwa, Keisuke Shigeta, Tsukasa Masuda, Yuto Baba, Ryuichi Mizuno, Mototsugu Oya

**Affiliations:** 1 Department of Urology, Keio University School of Medicine, Tokyo, Japan; 2 Department of Urology, St. Marianna University School of Medicine, 2-16-1 Sugao, Miyamae-ku, Kawasaki-shi, Kanagawa 216-8511 Japan

**Keywords:** Cancer, Urology

## Abstract

Limited information is currently available on predictors of upper tract urothelial carcinoma (UTUC) recurrence in non-muscle-invasive bladder cancer (NMIBC) patients according to smoking history, although smoking probably contributes to urothelial carcinogenesis. Therefore, the present study aimed to identify independent predictors of UTUC recurrence in all patients and those with a smoking history. Our study population comprised 1190 NMIBC patients who underwent transurethral resection of bladder tumor. UTUC developed in 43 patients during the follow-up. A history of bacillus Calmette-Guérin (BCG) therapy was independently associated with a lower incidence of UTUC (HR = 0.43; *P* = 0.011). In a subgroup of NMIBC patients with a smoking history, concomitant carcinoma in situ (CIS) and a lower urinary pH (< 6) were independently associated with a higher incidence of UTUC recurrence (HR = 3.34, *P* = 0.006 and HR = 3.73, *P* = 0.008, respectively). Among patients with a longer smoking duration (≥ 20 years) or larger smoking intensity (≥ 20 cigarettes per day), those with lower urinary pH (< 6) had a significantly higher UTUC recurrence rate than their counterparts. These results suggest that BCG instillation may prevent UTUC recurrence in NMIBC patients, while a lower urinary pH and concomitant CIS increase the risk of UTUC recurrence in those with a smoking history.

## Introduction

The incidence of upper tract urothelial carcinoma (UTUC) in patients with non-muscle-invasive bladder cancer (NMIBC) after transurethral resection was previously reported to be 0.5–6%^1–4^. The detection of UTUC recurrence in the early stage is considered to be important because it is more invasive than bladder cancer: 60% of UTUC are invasive at diagnosis, in contrast to only 15% of bladder tumors^5,6^. Approximately 10% of high-risk NMIBC patients developed UTUC, 20% of whom died from it^1,7^. Consequently, most NMIBC patients have received surveillance for UTUC development and bladder recurrence.

Previous studies on UTUC recurrence identified risk factors including the occurrence of intravesical recurrence, the presence of the vesicoureteral reflux (VUR), and failed intravesical chemotherapy^1,7–9^. Moreover, smoking has been shown to contribute to the carcinogenesis of UTUC^5^. Specifically, tobacco use increases the relative risk of UTUC to 2.5–7^10^. McLaughlin et al. showed that the estimated risk of UTUC ranged between 2.4 with the consumption of less than 20 cigarettes per day and 4.8 with the consumption of more than 40 cigarettes per day^11^. Therefore, smoking appears to be an important risk factor for UTUC development.

Urinary pH was recently found to be strongly associated with bladder carcinogenesis. An in vitro study showed that urinary pH strongly influenced the presence of aromatic amines from cigarette smoke and further metabolism to bind to DNA under acidic conditions^12^. Clinically, a consistent acidic urinary pH ≤ 6 increased the risk of bladder cancer, particularly in current smokers^13^. These findings indicate that acidic urine in smokers is associated with urothelial carcinogenesis. However, to the best of our knowledge, the relationships between the recurrence of UTUC and smoking status/urinary pH have not yet been examined in NMIBC patients. Accordingly, the aim of the present study was to clarify these relationships.

## Results

### Patient characteristics

The mean age of 1190 patients was 68.5 years and the mean follow-up was 67.3 months. After surgery, UTUC recurrence was identified in 43 cases (3.6%). Table 1 shows the clinicopathological characteristics of patients with and without UTUC recurrence. Among all patients, significant differences were observed in clinicopathological parameters, except for a history of bacillus Calmette-Guérin (BCG) therapy, between those with and without UTUC (Table 1). Among patients with a smoking history, only carcinoma in situ (CIS) and urinary pH were significantly different between the two groups.

**Table 1:** Characteristics of all patients and those with a smoking history according to UTUC recurrence after TURBT.

Characteristics	UTUC recurrence in all Pts			UTUC recurrence in Pts with a smoking history		
	No	Yes	*P* value	No	Yes	*P* value
**Age**			0.548			0.519
Younger than 70 years	587	20		360	13	
70 years or older	560	23		301	14	
**Sex**			0.831			0.878
Male	948	35		617	25	
Female	199	8		44	2	
**Smoking history**			0.501			
Positive	661	27				
Negative	486	16				
**Grade**			0.462			0.561
G1–2	704	24		404	15	
G3	443	19		257	12	
**Pathological T stage**			0.780			0.812
Ta	777	30		455	18	
T1	370	13		206	9	
**Multiple**			0.258			0.856
Yes	566	25		331	14	
No	581	18		330	13	
**Concomitant CIS**			0.197			0.007
Yes	138	8		65	7	
No	1009	35		596	20	
**Intravesical chemotherapy**			0.140			0.098
Yes	100	1		61	0	
No	1047	42		600	27	
**Urinary pH level**			0.056			0.003
Less than 6	577	28		348	22	
6 or more	570	15		313	5	
**BCG**			0.029			0.386
Yes	595	15		350	12	
No	552	28		311	15	

UTUC, upper urinary tract urothelial carcinoma; Pts, patients; No, number; CIS, carcinoma in situ.

### Predictors of UTUC recurrence in all NMIBC patients

**Figure 1:**
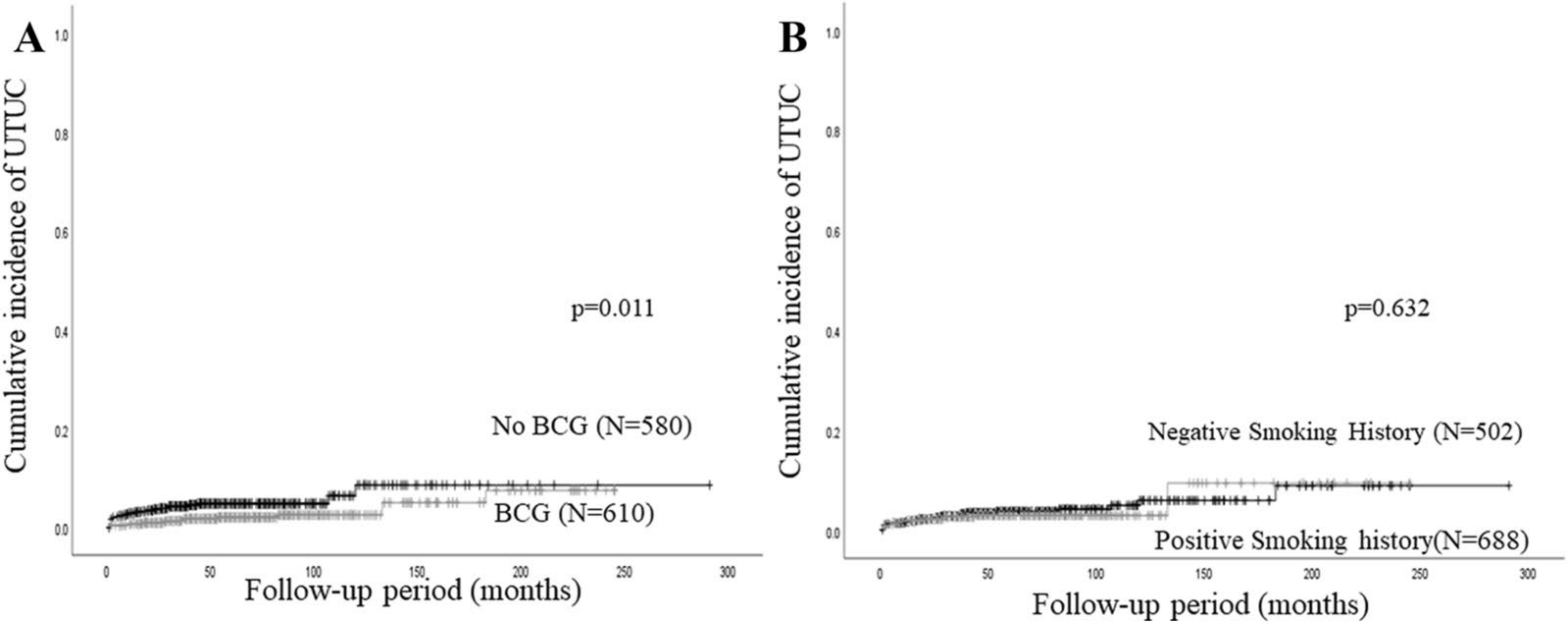
Cumulative incidence of UTUC recurrence in all NMIBC patients stratified by a history of BCG therapy (**A**) and the presence or absence of a smoking history (**B**).

The univariate Cox regression analysis identified BCG therapy as a significant predictor of UTUC recurrence in all NMIBC patients (Table 2). Furthermore, BCG therapy was independently associated with a lower incidence of UTUC recurrence (hazard ratio: HR = 0.43; *P* = 0.011). The 5-year UTUC recurrence rate was 4.9% in NMIBC patients who did not receive BCG therapy, which was significantly higher than in NMIBC patients treated with BCG therapy (5-year recurrence rate; 2.1%, *P* = 0.011, Fig. 1A). As shown in Fig. 1B, no significant difference was observed in UTUC recurrence between NMIBC patients with and without a smoking history.

**Table 2:** Relationships between clinicopathological factors and upper urinary tract recurrence after TURBT in all patients and those with a smoking history.

	All Pts				Pts with a smoking history			
	Univariate		Multivariate		Univariate		Multivariate	
	HR (95%CI)	*P* value	HR (95%CI)	*P* value	HR (95%CI)	*P* value	HR (95%CI)	*P* value
**Age**		0.255				0.252		
< 70 years	Ref				Ref			
≥ 70 years	1.43 (0.77–2.63)				1.51 (0.70–3.26)			
**Sex**		0.762				0.793		
Male	Ref				Ref			
Female	1.13 (0.52–2.43)				1.21 (0.29–5.13)			
**Smoking history**		0.632						
Negative	Ref							
Positive	1.16 (0.63–2.17)							
**Grade**		0.379				0.435		
G1–2	Ref				Ref			
G3	1.31 (0.72–2.39)				1.35 (0.63–2.89)			
**pT stage**		0.876				0.780		
Ta	Ref				Ref			
T1	0.95 (0.50–1.82)				1.12 (0.50–2.50)			
**Multiple**		0.219				0.798		
No	Ref				Ref			
Yes	1.47 (0.80–2.70)				1.10 (0.52–2.33)			
**Concomitant CIS**		0.194				0.006		0.006
No	Ref				Ref		Ref	
Yes	1.67 (0.77–3.57)				3.33 (1.41–7.69)		3.34 (1.41–7.93)	
**Chemotherapy**		0.189				0.288		
No	Ref				Ref			
Yes	0.26 (0.04–1.92)				0.04 (0.00–14.28)			
**BCG**		0.011		0.011		0.242		
No	Ref		Ref		Ref			
Yes	0.44 (0.23–0.83)		0.43 (0.23–0.83)		0.63 (0.29–1.37)			
**Urinary pH level**		0.072				0.008		0.008
6.0 or greater	Ref				Ref		Ref	
Less than 6	1.79 (0.95–3.33)				3.70 (1.41–10.0)		3.73 (1.41–9.84)	

UTUC, upper urinary tract urothelial carcinoma; Pts, Patients; HR, hazard ratio; CI, confidence interval; CIS, carcinoma in situ.

### Predictors of UTUC recurrence in NMIBC patients with a smoking history

**Figure 2:**
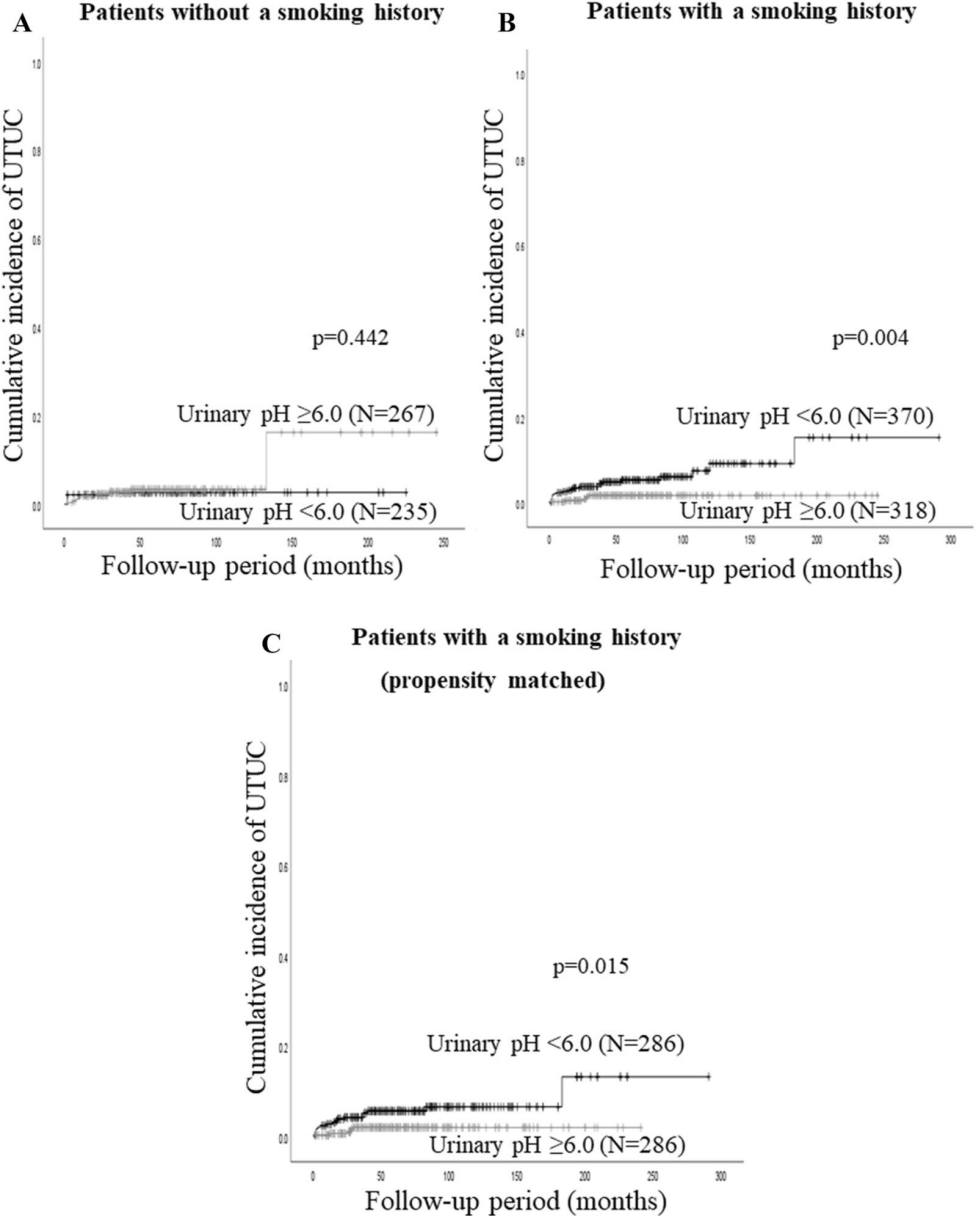
Cumulative incidence of UTUC recurrence stratified by urinary pH levels in (**A**) NMIBC patients without a smoking history, (**B**) those with a smoking history before a propensity matching analysis and (**C**) those with a smoking history after a propensity matching analysis.

The univariate Cox regression analysis identified concomitant CIS and the urinary pH level as significant predictors of UTUC recurrence in NMIBC patients with a smoking history (Table 2). Furthermore, concomitant CIS and a urinary pH level of less than 6 were independently associated with a higher incidence of UTUC recurrence (HR = 3.34, *P* = 0.006 and HR = 3.73, *P* = 0.008, respectively). As shown in Fig. 2A, in a subgroup of NMIBC patients without a smoking history, no significant difference was observed in UTUC recurrence between patients with a urinary pH level of equal to or more than 6 and those with a urinary pH level of less than 6. However, in a subgroup of NMIBC patients with a smoking history, those with a urinary pH level of less than 6 had a significantly higher incidence of UTUC recurrence than their counterparts (*P* = 0.004, Fig, 2B). In patients with a smoking history, 5-year UTUC recurrence rates were 5.4% and 2.1% in NMIBC patients with a urinary pH level of less than 6 and equal to or more than 6, respectively.

**Figure 3:**
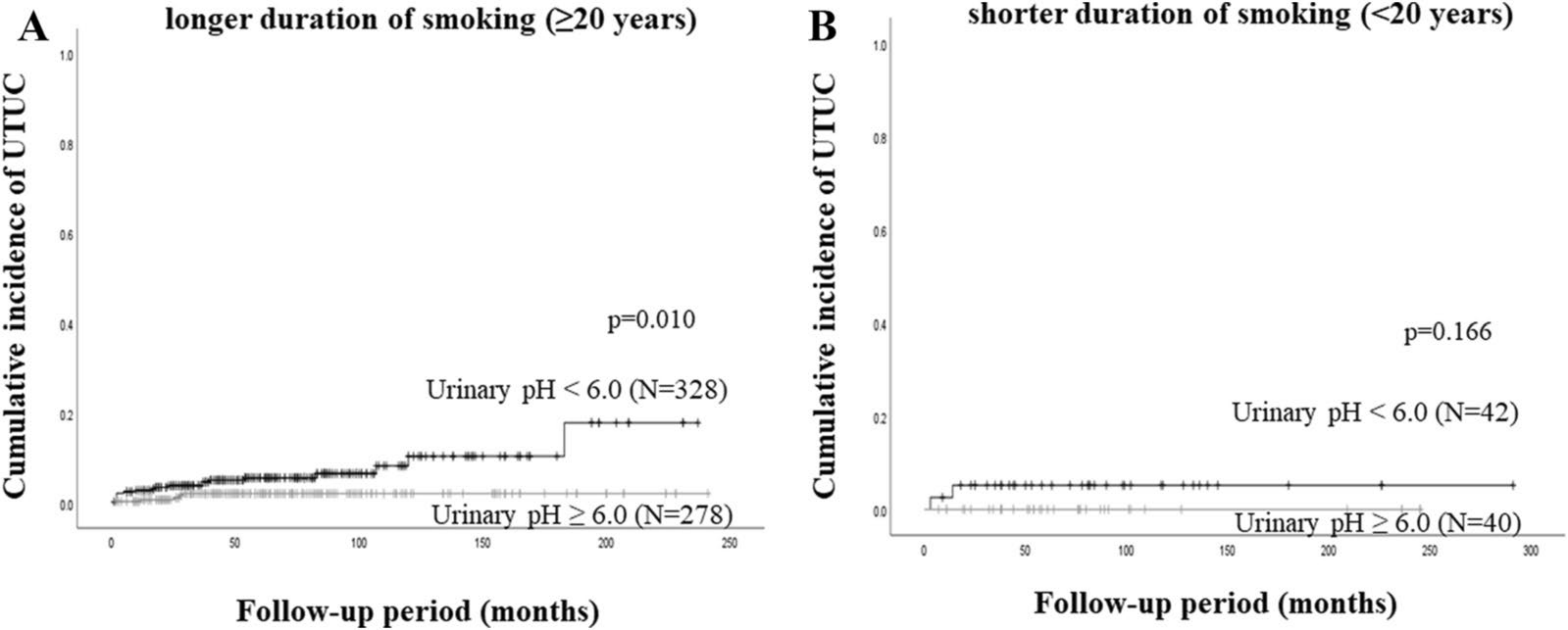
Cumulative incidence of UTUC recurrence stratified by urinary pH levels in patients who had a smoking history and (**A**) a longer duration of smoking (≥ 20 years) or (**B**) shorter duration of smoking (< 20 years).

Among NMIBC patients with a longer smoking duration (≥ 20 years), the 5-year UTUC recurrence rate was 5.6% in patients with a urinary pH level of less than 6, which was significantly higher than that in those with a urinary pH level of equal to or more than 6 (2.1%, *P* = 0.010, Fig. 3A). On the other hand, among patients with a shorter smoking duration (< 20 years), no significant difference in UTUC recurrence was found between the two groups (Fig. 3B).

**Figure 4:**
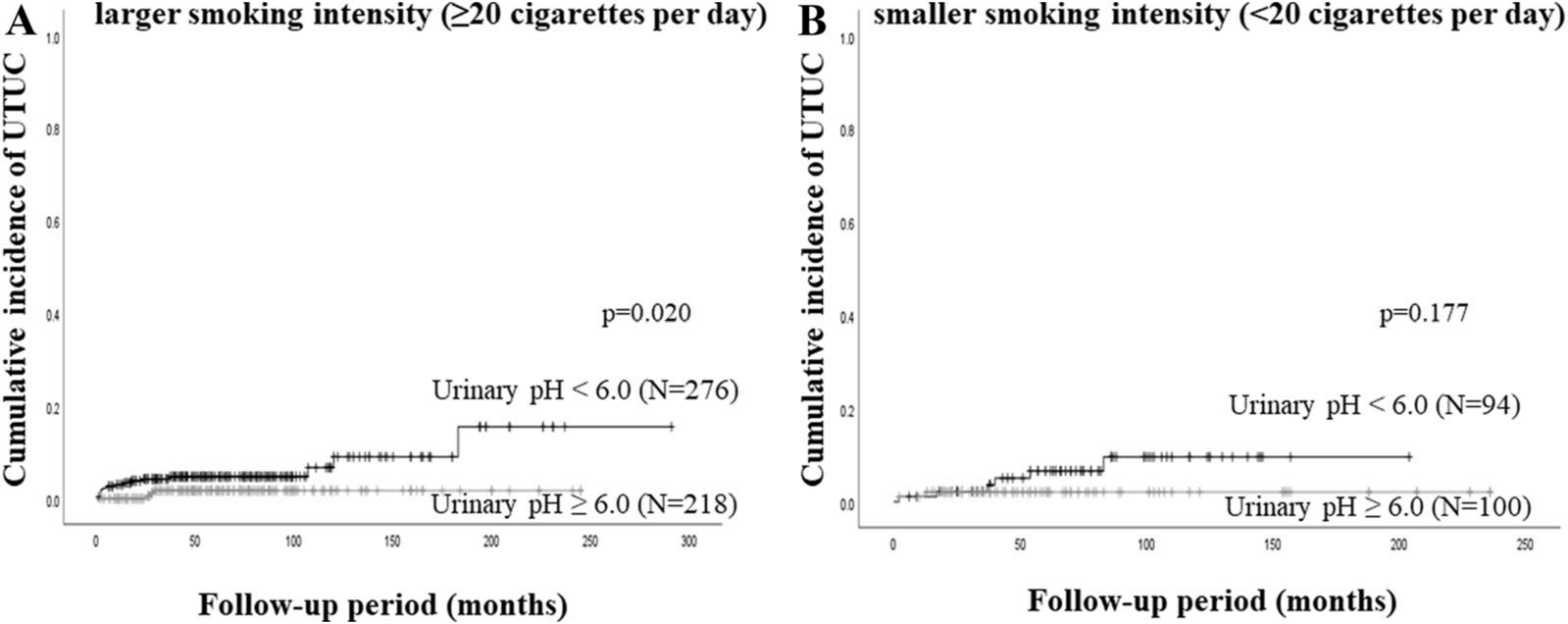
Cumulative incidence of UTUC recurrence stratified by urinary pH levels in patients who had a smoking history and (**A**) larger smoking intensity (≥ 20 cigarettes per day) or (**B**) smaller smoking intensity (< 20 cigarettes per day).

Similarly, among NMIBC patients with a larger smoking intensity (≥ 20 cigarettes per day), the 5-year UTUC recurrence rate was 4.8% in patients with a urinary pH level of less than 6, which was significantly higher than that in those with a urinary pH level of equal to or more than 6 (1.7%, *P* = 0.020, Fig. 4A). Meanwhile, no significant difference in UTUC recurrence between the two groups was observed in those with a smoking history and smaller smoking intensity (< 20 cigarettes per day) (Fig. 4B).

Propensity score matching yielded 286 patients in each group. Patients were similarly matched for all clinicopathological characteristics (Table 3). After the propensity matching analysis, in a subgroup of NMIBC patients with a positive smoking history, the 5-year UTUC recurrence rate was 5.7% in patients with a urinary pH level of less than 6, which was significantly higher than that in those with a urinary pH level of equal to or more than 6 (2.1%, *P* = 0.015, Fig. 2C).

**Table 3:** Characteristics of all patients and those with a smoking history according to urinary pH levels after TURBT.

	All Pts			Pts with a smoking history			Pts with a smoking history (propensity matched)		
	No. of pH < 6.0	No. of pH ≥ 6.0	*P* value	No. of pH < 6.0	No. of pH ≥ 6.0	*P* value	No. of pH < 6.0	No. of pH ≥ 6.0	*P* value
**Age**			0.219			0.025			0.353
Younger than 70 years	298	309		186	187		168	157	
70 years or older	307	276		184	131		118	129	
**Sex**			0.001			0.007			0.716
Male	521	462		354	288		271	269	
Female	84	123		16	30		15	17	
**Smoking history**			0.018			0.240			0.674
Positive	370	318		212	168		160	155	
Negative	235	267		158	150		126	131	
**Grade**			0.644			0.296			0.551
G1–2	374	354		232	187		173	166	
G3	231	231		138	131		113	120	
**Pathological T stage**			0.304			0.918			0.652
Ta	402	405		255	218		194	199	
T1	203	180		115	100		92	87	
**Multiple**			0.387			0.080			0.802
Yes	293	298		197	148		139	136	
No	312	287		173	170		147	150	
**Concomitant CIS**			0.568			0.749			0.260
Yes	71	75		40	32		24	32	
No	534	510		330	286		262	254	
**Intravesical chemotherapy**			0.111			0.958			0.771
Yes	59	42		33	28		25	27	
No	546	543		337	290		261	259	
**BCG**			0.919			0.573			0.615
Yes	311	299		191	171		154	148	
No	294	286		179	147		132	138	

NMIBC, non-muscle invasive bladder cancer; Pts, patients; No, number; CIS, carcinoma in situ; Chemo, chemotherapy.

## Discussion

Previous studies reported the vital role of smoking in urothelial carcinogenesis^5^; however, other factor may also contribute to it because not all smokers have urothelial malignancies. Accordingly, the present study clarified the relationship between UTUC recurrence and clinicopathological parameters in NMIBC patients according to a smoking history. In all patients, a history of BCG therapy was identified as a significant predictor of UTUC recurrence, while in those with a positive smoking history, a lower urinary pH and concomitant CIS increased the risk of UTUC recurrence. These results suggest that BCG therapy may prevent UTUC in NMIBC patients. Meanwhile, acidic urine and concomitant CIS appeared to increase the risk of UTUC recurrence in patients with a smoking history.

The multifocality and frequent recurrences are well-known characteristics of urothelial cancer. There are currently the two main concepts which have been proposed to explain this phenomenon^14^. The first hypothesis is intraluminal seeding theory, in which the multifocality or recurrence of urothelial carcinoma occurs due to the release of tumor cells from the primary lesion and the implantation of tumor cells at different sites of the urothelium. Several clinical outcomes suggest the dissemination theory. The risk of UTUC after transurethral resection of bladder tumor (TURBT) was previously reported to be 0.5–6%, which is markedly lower than the risk of bladder cancer after nephroureterectomy for UTUC (30–40%)^1–4,7,8,15^. However, the risk of UTUC has been shown to increase by 15- to 22-fold if patients have VUR^16,17^. Therefore, local control, including intravesical treatments, plays an important role in preventing dissemination from the bladder. Regarding UTUC development in NMIBC patients, previous studies identified several risk factors, including the occurrence of intravesical recurrence, the presence of VUR, and failed intravesical chemotherapy^1,7,8^. The present study showed that BCG instillation was an independent factor for UTUC in all patients, which was supported by the first hypothesis described above, *i.e*. the mechanism of dissemination.

The second hypothesis postulates that multiple cells become initiated or partially transformed as a result of carcinogenic insults and acquire independent genetic alternations. Therefore, the detection and exclusion of carcinogens, such as aromatic amines, are essential for preventing the development of urothelial carcinoma. In in vitro studies, aromatic amines were found to be affected by urinary pH^18^. Specifically, the rapid hydrolyzation of the N-glucuronide of N-acetyl-benzidine and further metabolism to bind to DNA were observed under acidic conditions^19,20^. Furthermore, the half-life of 4-aminobiphenyl (ABP) N-glucuronide conjugates before being hydrolyzed was 11 min at pH 5.5 and 3 h at pH 7.4^21^. Bois et al*.* also reported that urinary pH was a strong contributor to interindividual variations in the DNA binding of ABP in the bladder^22^. Clinically, previous studies reported that a consistent acidic urinary pH increased the risk of bladder cancer^13^ as well as bladder recurrence in UTUC^23^. Collectively, these findings indicate that acidic urine is a key factor for urothelial carcinogenesis and recurrence through the activation of aromatic amines derived from cigarettes. The subgroup analysis of smokers in the present study revealed a significant difference in the incidence of UTUC between the higher and lower urinary pH groups in patients with a larger smoking intensity or longer duration of smoking, but no significant differences between these groups in the counterparts. Moreover, after reducing the bias of multiplicity using propensity score matching, similar results were obtained. Taken together, urine pH appears to be important for UTUC recurrence in NMIBC patients with a smoking history.

NMIBC patients with CIS were previously reported to have a higher incidence of UTUC recurrence than those without it (21.2% vs 2.3%, *P* < 0.001)^24^. Schwartz et al. showed that the UTUC recurrence rate was 13% in NMIBC patients with CIS, which was significantly higher than that in those without CIS (3.1%)^25^. A recent study demonstrated that the smoke load (over 20 pack-year) increased the risk of recurrence and progression (HR = 1.019 and 1.034, *P* = 0.00004 and 0.00002, respectively) in NMIBC patients treated with BCG, suggesting that the smoke load reduces the efficacy of BCG therapy^26^. These findings appear to support our results showing that concomitant CIS, but not a history of BCG, is an independent predictor of UTUC recurrence in NMIBC patients with a positive smoking history.

Although the incidence of UTUC is relatively low^1,2,7,8,15^, most urologists perform UTUC surveillance for all NMIBC patients. Based on the present results, NMIBC patients may be divided into several risk groups based on smoking history. BCG may prevent UTUC recurrence in all NMIBC patients, while BCG and the inhibition of transcription factors induced in an acidic environment may effectively prevent UTUC recurrence in those with a smoking history. A recent study demonstrated that an acidic environment promoted tumor progression through the activation of sterol regulatory element-binding protein 2 (SREBP2)^27^, which was reported to function with p53^28^. A p53 mutation was detected in more cases of bladder cancer patients with a smoking history than in those without it^29^. Accordingly, further studies to investigate pH-regulated effectors of p53 in bladder cancer, such as SREBP2, might be needed to predict and prevent UTUC recurrence.

The present study has several limitations. Since it was performed in a retrospective manner, unknown sources of bias may exist. Accordingly, we performed a comparative analysis of the risk of UTUC in lower and higher pH NMIBC patients with a smoking history using propensity scoring to control for selection bias^30^. As aforementioned, there are several factors that alter urinary pH^31^, and patients with diseases or taking medication that affect urinary pH were excluded. The smoking status was self-reported, which may cause a recall bias. Regarding urinary pH measurements, the accuracy of the dipstick test is also a study limitation. The gold standard measurement of urinary pH is with an electrochemical pH meter. However, it is not clinically used due to its complexity and cost.

In conclusion, the results of the present study suggest that BCG instillation prevented UTUC recurrence in NMIBC patients. Acidic urine and concomitant CIS increase the risk of UTUC recurrence in NMIBC patients with a smoking history. Therefore, monitoring urine pH and modifications to pH for urine alkalization may benefit NMIBC patients with a positive smoking history.

## Methods

We retrospectively reviewed the medical records of patients who were surgically treated at Keio University Hospital, Saiseikai Central Hospital, and Saitama Medical University Hospital between 1995 and 2014. During this period, 1293 NMIBC patients underwent TURBT at these institutions. This study was approved by the Ethics Committee of Keio University Hospital, Saiseikai Central Hospital and Saitama Medical University Hospital, and was performed in accordance with the guidelines of human research. All subjects involved in the study provided their written informed consent according to the Declaration of Helsinki, as approved by the Ethics Committee of Keio University Hospital, Saiseikai Central Hospital and Saitama Medical University Hospital.

Patients with several diseases that alter urinary pH, including renal tubular acidosis, primary hyperparathyroidism, and urinary tract infection (UTI) were excluded from the present study^23^. Additionally, patients taking certain medications that affect urinary pH levels were excluded^23^. Accordingly, 4 patients with UTI and 3 being treated with medications that affect urinary pH during the perioperative period were excluded from this study. Also, 3 patients who were lost to the follow-up within 2 months of TURBT, 84 with a previous history of UTUC and 7 with synchronous UTUC, and 2 with insufficient information on their smoking status or urine analysis were excluded from this study. We ultimately analyzed 1190 patients in the present study. The histological types were pure urothelial carcinoma (UC) and UC with the other histological components in 1101 and 89 patients, respectively. Among the other concomitant histological components, squamous cell carcinoma (SCC) component alone, adenocarcinoma (AC) component alone, and both SCC and AC components were found in 14, 39, and 36 patients, respectively. Among them, 610 patients received induction BCG therapy scheduled for weekly administration for 6–8 weeks at a full dose of BCG in 40 ml of saline with retention for 1–2 h. Basically, intravesical BCG therapy was utilized in accordance with the current guideline^32^. Meanwhile, several patients did not receive it due to their unwillingness or the preference of the attending physicians.

Patients were subjected to urine cytology and cystoscopy every 3 months for 2 years after TURBT, every 6 months for the next 3 years, and every 6 to 12 months thereafter. Computerized tomography, magnetic resonance imaging, and/or excretory urograms were also performed every year for 5 years after TURBT and annually thereafter^33^.

We measured urinary pH using Uriflet 9UB (Menarini Diagnostics, Florence, Italy), a widely used dipstick in Japan, and Autin MAX™ AX-4030, an automated urine test-strip analyzer^34^. Urinary pH was defined as the median of at least 3 consecutive measurements within 2 months of TURBT. Voided urine samples were collected from the second or third micturition of the morning.

Data on self-reported cigarette smoking statuses were obtained in an interview with attending physicians as part of the patient history at the initial consultation. These data were confirmed by a resident or fellow in a secondary interview at TURBT^33^. In the present study, NMIBC patients who had smoked at least 100 cigarettes in their lifetime were defined as patients with a smoking history. Among 688 patients with a smoking history, 380 current and 308 former smokers who had quit smoking at the time of the interview were included.

We analyzed relationships between UTUC recurrence and clinicopathological parameters, including the smoking status and urinary pH, using the chi-squared test. The incidence of UTUC was estimated using the Kaplan–Meier method and analyzed with the Log-rank test. A multivariate analysis was performed using the Cox proportional hazards model. Variables that were marginally significant at a level of *P* < 0.10 in the univariate analysis were selected in the multivariate model. Therefore, we included BCG and urinary pH in all patients and urinary pH and concomitant CIS in those with a positive smoking history in the final multivariate model. We performed propensity score analyses to compare the risk of UTUC between the low and high pH groups with similar clinicopathological parameters in NMIBC patients with a smoking history. Propensity scores were estimated using a multivariate logistic regression method, in which variables included age, sex, the smoking status, grade, T stage, multiplicity, CIS, intravesical chemotherapy, and BCG.

Ideal cut-off values for variables were calculated by applying a receiver-operating curve analysis to test all possible cut-offs that may discriminate between patients with or without UTUC development^35^. A urinary pH level of 6.0 was defined as the cut-off point in the present study. The level of significance was set at *P* < 0.05. These analyses were performed with the SPSS statistical software package, version 26.0 (SPSS: An IBM Company, Chicago, IL).

## Data Availability

The datasets generated during the present study are available from the corresponding author upon reasonable request.
